# Patent Foramen Ovale in Cryptogenic Stroke and Migraine with Aura: Does Size Matter?

**DOI:** 10.7759/cureus.3213

**Published:** 2018-08-27

**Authors:** Saeed S Sadrameli, Rajan R Gadhia, Rasadul Kabir, John J Volpi

**Affiliations:** 1 Neurosurgery, Houston Methodist Neurological Institute, Houston, USA; 2 Neurology, Houston Methodist Neurological Institute, Houston, USA; 3 Radiology, Houston Methodist Neurological Institute, Houston, USA

**Keywords:** patent foramen ovale, transient ischemic attack, transcranial doppler, migraine

## Abstract

Introduction: There is an association between cryptogenic strokes and patent foramen ovale (PFO), as well as between migraines with aura and PFO. The purpose of the current study was to compare shunt characteristics in the stroke and migraine populations.

Methods: We retrospectively evaluated the degree of the shunt in 68 consecutive patients with cryptogenic stroke (n=33) or migraines with aura (n=35) evaluated in a single transcranial Doppler laboratory. All patients underwent an intravenous injection of agitated saline, followed by the insonation of the middle cerebral artery to determine the degree of the right-to-left shunt. We graded the shunt size according to the number of emboli: Grade I, none; Grade II, 1-10; Grade III, 11-100; and Grade IV, >100. Grades I and II were considered low-grade shunts, and Grades III and IV were considered high-grade.

Results: In the 14-month study period, we found 31 high-grade shunts and 37 low-grade shunts. Among migraines with aura patients, 27 (77%) had high-grade shunts, whereas only 4 patients (12%) with cryptogenic stroke had high-grade shunts. These percentages were significantly different between groups (Fisher’s exact test, p<0.0001).

Conclusions: In a standardized laboratory using uniform methods, we found a significant difference in shunt size associated with PFO between cryptogenic stroke and migraine with aura patients. We hypothesize that in migraines with aura, venous admixture with arterial blood is the main mechanism by which PFO contributes to the condition. In contrast, cryptogenic strokes associated with PFO are more likely to arise from an atrial septal clot within the PFO space.

## Introduction

An association exists between cryptogenic stroke and patent foramen ovale (PFO), as well as between migraines with aura and PFO. Previously, these relationships have been described as the presence or absence of a right-to-left shunt. However, little research has been done comparing shunt characteristics (such as size) in different disease populations. In the modern era of stroke care, the management of PFO has really taken a spotlight. The initial trials in 2012-2013 investigating the role of PFO closure in the reduction of recurrent embolic stroke failed to show any benefit over medical therapy [1-3]. However, looking at the recent trials published in the New England Journal of Medicine (NEJM), the pendulum has swung towards a consideration of the benefits of intervention in patients who otherwise would have been managed medically [4-6].

Our review of the literature revealed a handful of randomized, controlled clinical trials, which have investigated the efficacy of PFO closure (as suggested by meta-analyses and data reviews) in patients with a transient ischemic attack (TIA) or cryptogenic stroke [7-8]. In March 2012, the CLOSURE I investigators reported the results of their randomized, controlled trial evaluating the possible superiority of PFO closure with the STARflex device (NMT Medical, Boston, MA, USA) with the addition of antiplatelet therapy, over medical therapy (warfarin, aspirin, or both) alone in decreasing the risk of recurrent stroke or TIA. PFO closure, however, did not provide any added benefit in preventing recurrent stroke or TIA [3]. The following year, two separate groups reported their results comparing PFO closure to medical management. The Randomized Evaluation of Recurrent Stroke Comparing PFO Closure to Established Current Standard of Care Treatment (RESPECT) group conducted a prospective, multicenter, randomized controlled trial comparing the efficacy of PFO closure to medical therapy alone in preventing recurrent ischemic stroke or early death. Similarly, the Clinical Trial Comparing Percutaneous Closure of Patent Foramen Ovale Using the Amplatzer PFO Occluder with Medical Treatment in Patients with Cryptogenic Embolism (PC trial) investigators evaluated the possible superiority of percutaneous closure of PFO over medical therapy in the secondary prevention of cryptogenic embolism. Both trials found no added benefit with PFO closure on the primary endpoint when compared to medical therapy alone [1-2]. As is often the case within the field, a study designed with appropriate inclusion and exclusion criteria can mean the difference between the significance or failure of a trial. The recent studies published late in 2017 suggest that the treatment of PFO could potentially reduce the risk of recurrent embolic strokes [4,6]. While one should critically analyze the previous results and the outcomes of recent trials, in a recent editorial published in the NEJM, Ropper concludes that the possible benefits of PFO closure in the reduction of embolic strokes are contingent on the characteristics of PFO [9]. In this study, we aimed to focus our attention on PFO features, such as size or grade of the shunt, and investigate possible correlations between shunt size and the incidence of migraines with aura or embolic cryptogenic stroke.

When critically analyzing the studies of PFO directly comparing shunt size within stroke or migraine populations, a clear link between the size of shunt and pathogenicity has not been established [10]. Much of the focus in previous studies was on the association between PFO size and various etiologies of stroke, rather than the association between PFO size in migraine and stroke patients. One group found that medium and large PFOs were more prevalent in patients with a cryptogenic stroke than in patients with a stroke of other, known etiologies. In addition, they found that intracranial imaging findings suggestive of embolic infarcts were more frequent in stroke patients with a large PFO than in those patients with a small PFO [11]. Others have reported no association between migraines and the presence of a PFO and, to take it a step further, no association between migraine headaches and the grade of shunt detected by either transthoracic echocardiography or transcranial Doppler (TCD) [12]. Several studies investigating the presence of an atrial septal aneurysm in addition to PFO have shown a higher rate of cryptogenic stroke, and recurrent stroke, with the combination when compared with PFO without associated atrial septal aneurysm [13-14]. Most of the existing literature lacks subgroup analyses according to shunt size. However, a closer examination of PFO subgroups in the Cryptogenic Stroke Study (PICSS) suggests that the two-year recurrent stroke or death rates were higher in patients with a small PFO than in those with a large PFO [10]. Furthermore, methods of PFO evaluation and shunt grading varied considerably in previous studies, which limits the ability to perform a valid comparison between these studies.

To address the gaps in the existing literature, we sought to determine whether patients with PFO and migraines with aura have characteristic shunt sizes. We also sought to evaluate the degree of shunt in patients with cryptogenic stroke and to compare the shunt sizes in these two patient populations.

## Materials and methods

In this study, we retrospectively evaluated the degree of shunt in 68 consecutive patients with cryptogenic stroke (n=33) or migraines with aura (n=35) who were evaluated in a single TCD laboratory between January 2014 and July 2015. All patients had either a remote or a recent history of stroke or migraines with aura. To perform the bubble study confirmation and grading of a shunt, a peripheral intravenous catheter was inserted in all patients. A total of 10 mL agitated saline was prepared by mixing 9 mL saline and 1 mL air by repeated transfer between two 10 mL syringes connected by a three-way stopcock. Agitated saline was then injected intravenously and insonation of the middle cerebral artery was performed to determine the shunt size. We graded the size of shunt according to the number of emboli detected: Grade I, none; Grade II, 1-10; Grade III, 11-100; and Grade IV, > 100. Grades I and II were classified as low-grade shunts, whereas Grades III and IV were classified as high-grade.

Fisher’s exact test was used to compare the percentages of patients with high-grade shunts in the two populations. A p-value <0.05 was considered statistically significant.

## Results

**Figure 1:**
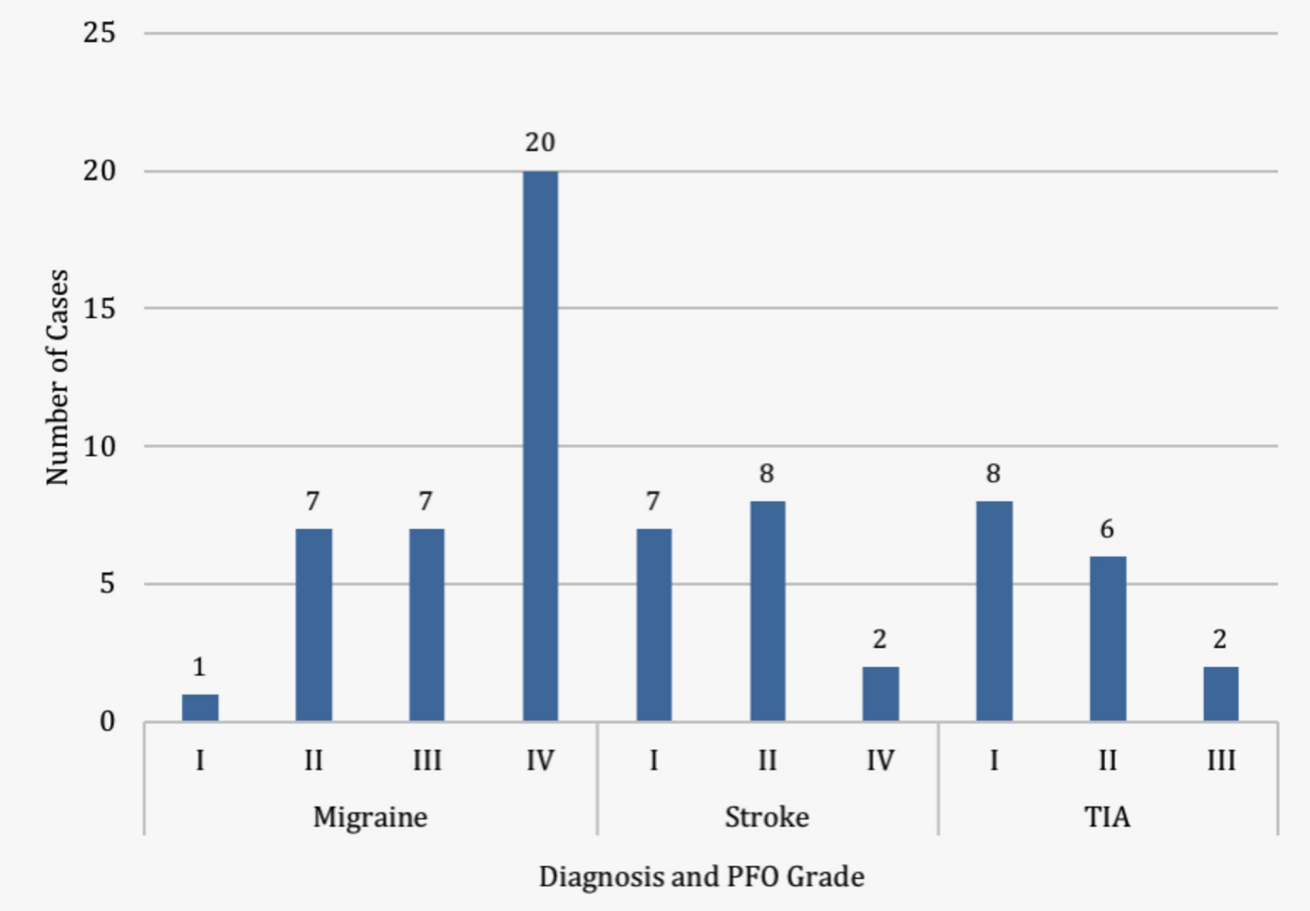
Number of low- and high-grade patent foramen ovale (PFOs) in patients with migraine with aura, stroke, and transient ischemic attack (TIA)

**Figure 2:**
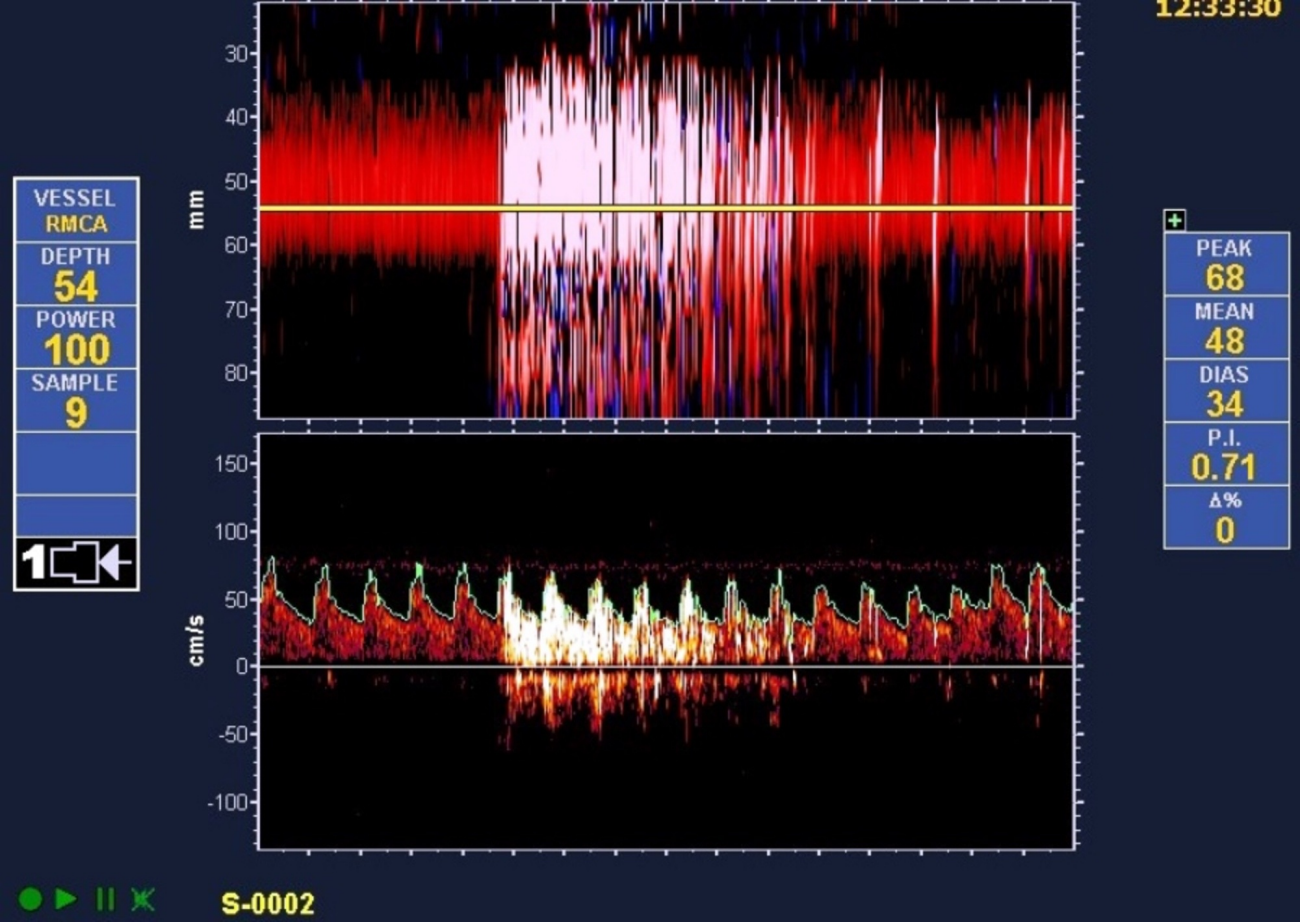
After an injection of agitated saline: transcranial Doppler of the right middle cerebral artery in a patient with a complex migraine with aura

**Figure 3:**
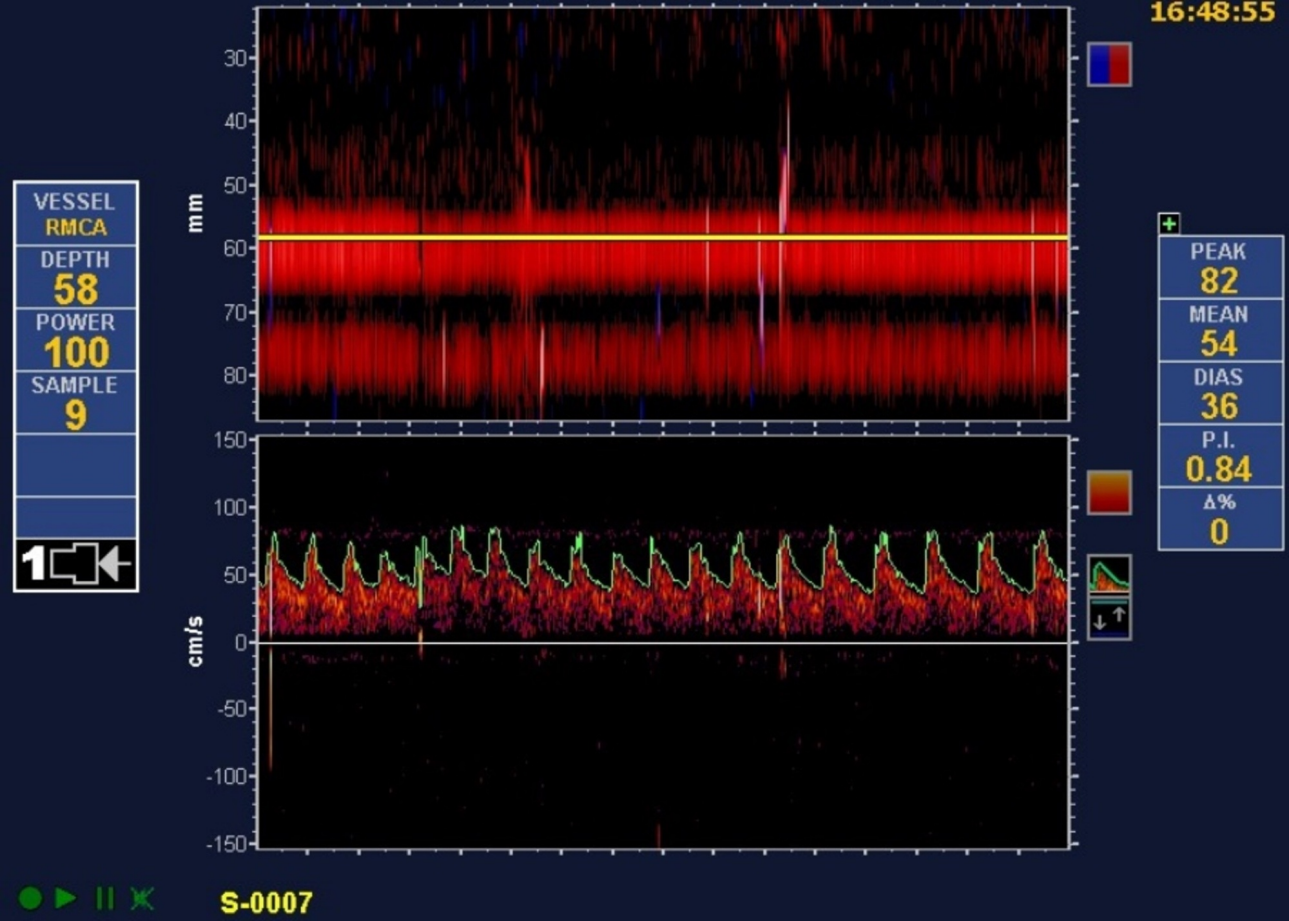
After an injection of agitated saline: transcranial Doppler of the right middle cerebral artery in a patient with a cryptogenic stroke

In the 14-month period of observation, we found 31 high-grade shunts and 37 low-grade shunts. Among the 35 migraine with aura patients, 27 (77%) had high-grade shunts. Among the 33 cryptogenic stroke patients, only four (12%) were noted to have high-grade shunts (Figure 1). High-grade shunts were significantly more common in patients with migraines with aura than in those with a cryptogenic stroke (p<0.0001). Our study also demonstrated a substantial difference in the degree of shunt size between patients with cryptogenic stroke and patients with migraines with aura, with the latter associated with a larger shunt size (Figures 2-3).

## Discussion

PFO management has changed dramatically in the past year. It was notorious for inconsistency in patient management, primarily due to differences in opinions on appropriate treatment and secondary stroke prevention strategies. The question of whether PFO closure can prevent recurrent strokes in the setting of paradoxical embolism has now definitively been answered. Patient selection, and thus stroke work-up to determine the etiology of an infarct, remains an integral component of the equation. Differences in the definition of cryptogenic stroke, i.e., a stroke in the setting of PFO, as well as variability in inclusion and exclusion criteria between the large randomized studies, makes it difficult to make accurate predictions of high-risk PFO characteristics. We feel that shunt size, as graded by TCD, plays a significant role in the pathophysiology and PFO-attributable stroke risk.

When looking more in-depth at the migraine population, 60% of patients with a migraine with aura have a PFO, whereas the prevalence is 15% to 25% in the general population. Multiple mechanisms have been proposed to explain the possible causal relationship between PFO and migraines with aura [15]. We hypothesize that a larger PFO shunt grade translates to more neurotransmitter and venous deoxygenated hemoglobin contamination of the arterial vasculature, which, in turn, causes a neurovascular activation and abnormal vasoregulation corresponding to a typical migraine headache. Metabolites such as serotonin, nitric oxide, and kinins bypass the pulmonary circulation via the PFO, enter the systemic circulation, and cause an irritation of the trigeminal nerve and brain vasculature, thereby producing migraine headaches. Serotonin, for example, is released by platelet aggregation and is typically metabolized in the lungs by the monoamine oxidase enzyme. Studies have suggested a direct association between migraines and an increase in platelet activation and aggregation. Therefore, in the presence of PFO, excess serotonin in the blood is diverted away from the lungs and triggers a migraine [16-17].

Other studies have suggested a direct association between transient hypoxemia and migraine episodes. In patients with PFO, deoxygenated blood in the venous circulation bypasses the pulmonary vasculature and can cause transient hypoxemia. Hypoxemia in the appropriate setting (e.g., exercise or stress) can, in turn, lead to irritation trigeminal-innervated structures, typical unilateral headaches, and in rare but severe cases of vascular dysfunction, micro-infarctions in the brain [17-19].

In addition to its putative role in migraines with aura, PFO is also thought to play an important role in the etiology of cryptogenic strokes by facilitating paradoxical embolism. However, the characteristics of the right-to-left shunts associated with PFO have not yet correlated with the pathophysiology of different disorders. Recent trials emphasize the importance of physiological characteristics of PFO when treatment is being considered [9]. It is often misinterpreted that larger shunt grade and size (criteria dependent on the study used to define characteristics) correlates with a higher risk of stroke. Our study’s cohort found this to be in direct contradiction, as our migraine with aura patients had significantly larger shunt grades compared to our stroke subjects. Thus, with the newfound knowledge of long-term outcomes, in one of the three recently published studies, with 10-year follow-up data showing a 47% absolute stroke risk reduction in the PFO closure cohort, it is imperative that the conventional considerations of shunt characteristics not deter patients from being offered the potential benefit of closure.

Our study highlights that shunt size was significantly associated with the type of disease process, but in a way that contradicts most of the large randomized studies. In a single, standardized laboratory using uniform methods, we found a substantial difference in the degree of shunt size between patients with cryptogenic stroke and patients with migraines with aura. This suggests that PFO plays a different pathophysiologic role in the two conditions. We hypothesize that in migraines with aura, an admixture of venous blood with arterial blood is the main mechanism by which PFO contributes to the condition. In contrast, the pathophysiology of cryptogenic stroke is unlikely to involve an admixture of blood but is more likely to arise from a septal clot within the PFO space or an associated intra-atrial septal aneurysm. Further studies looking more uniformly at grading shunt size and other PFO characteristics are needed to yield more definite conclusions.

We realize our study has a considerable number of limitations. To begin, our sample size is too small to yield any significant generalizable conclusions. However, the significant disproportion between the two cohorts does make a compelling need for further large-scale studies to help better answer the question. In addition, baseline characteristics were not collected and could, in themselves, be responsible for the conclusions based on possible age, gender, or other vascular risk factor differences between the two cohorts. The only required modalities for the confirmation of PFO in these patients was transthoracic echocardiography, which we realize is certainly not the most specific confirmatory method. The addition of confirmation with transesophageal echocardiography, or cardiac magnetic resonance imaging (MRI), would be an important confirmatory way to assess PFO characteristics.

## Conclusions

In this study, we found high-grade (Grades III or IV) shunts in more than three-quarters of patients with a PFO and migraines with aura. In contrast, approximately 10% of patients with a PFO and cryptogenic ischemic stroke had a high-grade shunt. These findings have major implications for the management of patients with migraines with aura as compared with the management of individuals with cryptogenic strokes. When deciding between therapeutic strategies, such as PFO closure versus medical management, the notion is not necessarily valid that larger shunts are more concerning than smaller shunts in patients who present with cryptogenic strokes. The fundamental outcomes could thus be skewed given the exclusion of potential patients in whom a PFO was to blame for their previously diagnosed cryptogenic stroke. We argue that further studies are required to fully explore the etiology and pathophysiology of migraines with aura and cryptogenic ischemic strokes, specifically in those patients with PFO. Based on our limited, but compelling findings, we also believe that the exclusion of patients for potential PFO closure should not be based solely on the size of a shunt.

## References

[1] Meier B, Kalesan B, Mattle HP (2013). Percutaneous closure of patent foramen ovale in cryptogenic embolism. N Engl J Med.

[2] Carroll JD, Saver JL, Thaler DE (2013). Closure of patent foramen ovale versus medical therapy after cryptogenic stroke. N Engl J Med.

[3] Furlan AJ, Reisman M, Massaro J (2012). Closure or medical therapy for cryptogenic stroke with patent foramen ovale. N Engl J Med.

[4] Søndergaard L, Kasner SE, Rhodes JF (2017). Patent foramen ovale closure or antiplatelet therapy for cryptogenic stroke. N Engl J Med.

[5] Saver JL, Carroll JD, Thaler DE (2017). Long-term outcomes of patent foramen ovale closure or medical therapy after stroke. N Engl J Med.

[6] Mas J-L, Derumeaux G, Guillon B (2017). Patent foramen ovale closure or anticoagulation vs. antiplatelets after stroke. N Engl J Med.

[7] Kitsios GD, Dahabreh IJ, Dabrh AMA (2012). Patent foramen ovale closure and medical treatments for secondary stroke prevention: a systematic review of observational and randomized evidence. Stroke.

[8] Agarwal S, Bajaj NS, Kumbhani DJ, Tuzcu EM, Kapadia SR (2012). Meta-analysis of transcatheter closure versus medical therapy for patent foramen ovale in prevention of recurrent neurological events after presumed paradoxical embolism. JACC Cardiovasc Interv.

[9] Ropper AH (2017). Tipping point for patent foramen ovale closure. N Engl J Med.

[10] Homma S, Di Tullio MR, Sacco RL, Mihalatos D, Mandri GL, Mohr JP (1994). Characteristics of patent foramen ovale associated with cryptogenic stroke. A biplane transesophageal echocardiographic study. Stroke.

[11] Steiner MM, Di Tullio MR, Rundek T (1998). Patent foramen ovale size and embolic brain imaging findings among patients with ischemic stroke. Stroke.

[12] Garg P, Servoss SJ, Wu JC (2010). Lack of association between migraine headache and patent foramen ovale: results of a case-control study. Circulation.

[13] Mas JL, Arquizan C, Lamy C, Zuber M, Cabanes L, Derumeaux G, Coste J (2002). Recurrent cerebrovascular events associated with patent foramen ovale, atrial septal aneurysm, or both. ACC Current Journal Review.

[14] Cabanes L, Mas JL, Cohen A (1993). Atrial septal aneurysm and patent foramen ovale as risk factors for cryptogenic stroke in patients less than 55 years of age. A study using transesophageal echocardiography. Stroke.

[15] Sathasivam S, Sathasivam S (2013). Patent foramen ovale and migraine: what is the relationship between the two?. J Cardiol.

[16] Borgdorff P, Tangelder GJ (2012). Migraine: possible role of shear-induced platelet aggregation with serotonin release. Headache.

[17] Sharma A, Gheewala N, Silver P (2011). Role of patent foramen ovale in migraine etiology and treatment: a review. Echocardiography.

[18] Naqvi TZ, Rafie R, Daneshvar S (2010). Potential faces of patent foramen ovale (PFO PFO). Echocardiography.

[19] Tobis JM, Azarbal B (2005). Does patent foramen ovale promote cryptogenic stroke and migraine headache?. Tex Heart Inst J.

